# Implantation of leadless pacemaker beside abandoned end-of-life leadless pacemaker in a patient with tricuspid transcatheter edge-to-edge repair

**DOI:** 10.1016/j.hrcr.2025.07.028

**Published:** 2025-08-06

**Authors:** Omer Mamoun Mohamed, Ahmed Alqasem, Mossab Aljauid, Mohammed Bara Qattea, Muhammad Azam Shah, Mohammed Alshehri

**Affiliations:** 1 Department of Electrophysiology, King Salman Cardiac Center, King Fahad Medical City, Riyadh, Saudi Arabia; 2 Department of Echocardiography, King Salman Cardiac Center, King Fahad Medical City, Riyadh, Saudi Arabia

**Keywords:** Complete heart block, Leadless pacemaker implantation, Abandoned leadless pacemaker, Tricuspid transcatheter Edge-to-Edge Repair, Retrieval of leadless pacemaker

**Key Teaching Points**

• Abandoning an end-of-life leadless pacemaker (LPM) is a reasonable strategy for high-risk patients when retrieval carries high procedural risks.
• Multimodal imaging (transesophageal echocardiography and fluoroscopy) is crucial to guide safe LPM implantation across a repaired tricuspid valve.
• Follow-up with imaging, Holter monitoring, and device interrogation is essential to assess device-to-device interactions over time.
• Future studies should explore the safety and feasibility of retrieving chronically implanted LPM through tricuspid valves repaired using transcatheter edge-to-edge repair.

## Introduction

Leadless pacemaker (LPM) is the preferred pacing strategy for high-risk patients, including those with diabetes mellitus, end-stage renal disease, and valvular heart disease. It carries less risk of infective endocarditis and less impact on tricuspid valve (TV) integrity compared with conventional pacemakers.^1^ Previous case reports have demonstrated successful implantation of LPMs in patients with TV transcatheter edge-to-edge repair (TEER) through neo-orifices, guided by echocardiography.^2^^,^^3^ Moreover, studies have demonstrated the feasibility and safety of implanting multiple LPMs within the right ventricle (RV).^4^^,^^5^ This case report illustrates the implantation of an AVEIR VR LPM (Abbott) beside an abandoned end-of-life Micra VR LPM (Medtronic) through a repaired TV using TEER (TriClip, Abbott), guided by transesophageal echocardiography (TEE) and fluoroscopy.

## Case report

A 58-year-old woman known to have hypertension, diabetes mellitus, chronic hepatitis B virus, and end-stage renal disease managed with regular hemodialysis via arteriovenous fistula presented for evaluation. She had a Micra VR implanted in April 2018 for complete heart block. Over time, she developed progressive tricuspid regurgitation (TR) because of pulmonary hypertension and annular dilatation, resulting in torrential TR and recurrent right-sided heart failure. She underwent tricuspid TEER in October 2022, which successfully reduced TR to a mild-to-moderate severity.

**Figure 1:**
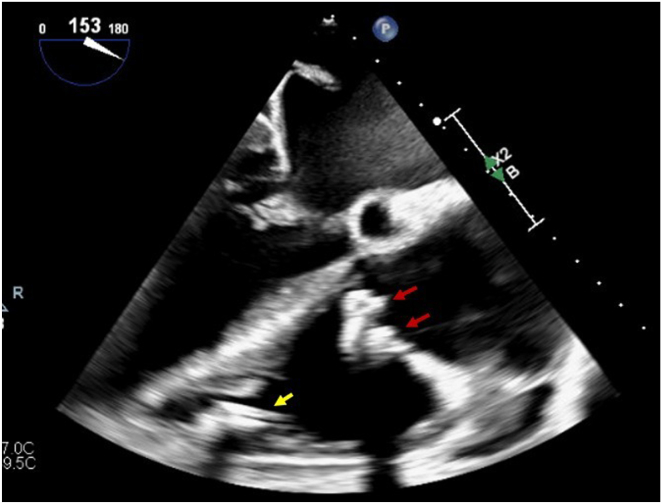
TEE mid-esophageal view at 153 degrees. Red arrows showed 2 TV clips, yellow arrow shows Micra VR. TEE = t*ransesophageal echocardiography*; TV = tricuspid valve.

During routine device interrogation, the Micra VR showed: Mode VVI at 60 bpm, battery at recommended replacement time, voltage 2.56 V (4 months), R-wave 17 mV, impedance 610 ohms, and capture threshold 1.13 V at 0.24 ms, with 92.2% ventricular pacing. After discussion with the patient and her family, the decision was made to implant a second LPM and deactivate the Micra VR, leaving it in place. Transthoracic echocardiography (TTE) showed preserved biventricular function and residual moderate TR. Preoperative TEE confirmed the position of 2 TV clips. (Figure 1).

**Figure 2:**
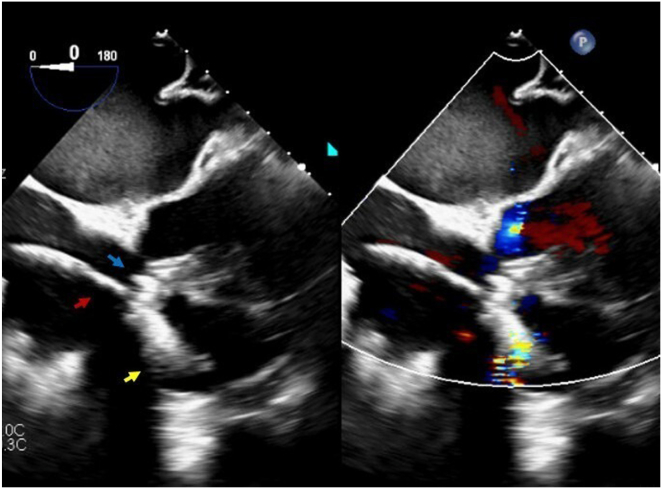
TEE mid-esophageal view at 0 degree. The *red arrow* shows the AVEIR VR delivery catheter crossing TV neo-orifice, the *blue arrow* shows TV clips, and the *yellow arrow* shows shadow from AVEIR VR inside the RV. RV = right ventricle; TEE = transesophageal echocardiography; TV = tricuspid valve.

We initiated the procedure under general anesthesia, and right femoral vein access was obtained using ultrasound guidance, and an 8Fr sheath was inserted. A super stiff Amplatz wire was advanced into the superior vena cava. The 8Fr sheath was upsized to 20Fr with dilators, and a 25Fr AVEIR VR introducer sheath was positioned at the inferior vena cava-right atrium junction. The delivery catheter was advanced to the RV through the tricuspid neo-orifice under fluoroscopic and TEE guidance (Figure 2).

**Figure 3:**
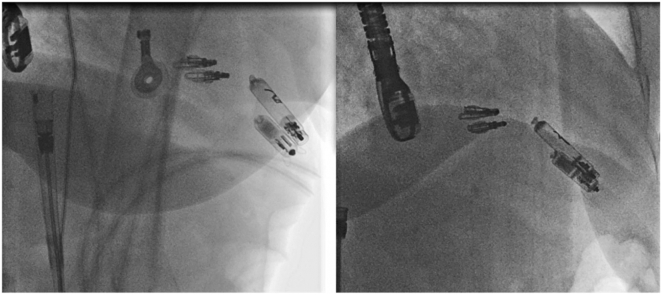
Fluoroscopy RAO 60 and 30-degree projections showing parallel devices where AVEIR VR above the depleted Micra VR. RAO = right anterior oblique.

Using the fluoroscopic right and left anterior oblique projections, the AVEIR VR was positioned in the RV mid-septum just above the abandoned Micra VR (Supplemental Videos 1 and 2). While positioning the AVEIR VR, the patient experienced a brief 5-second episode of asystole, which resolved spontaneously without requiring activation of transcutaneous pacing pads. The final position showed parallel alignment of both devices without mechanical interaction and acceptable screening parameters (Figure 3).

**Figure 4:**
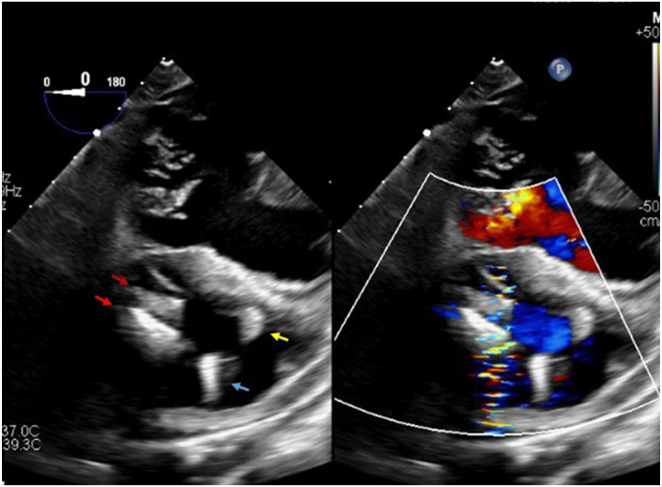
TEE mid-esophageal view at 0 degree. *Red arrows* showed TV clips, *blue arrow* showed part of depleted Micra VR, and *yellow arrow* showed part of AVEIR VR after deployment. TEE = transesophageal echocardiography; TV = tricuspid valve.

The AVEIR VR was successfully deployed with satisfactory sensing, impedance, and capture threshold, while the Micra VR was deactivated. Post-implant TEE showed an intact tricuspid apparatus and unchanged TR (Figure 4). The patient was monitored for 24 hours, recovered smoothly, ensuring no device-to-device interaction, and then discharged home the following day in stable condition.

**Figure 5:**
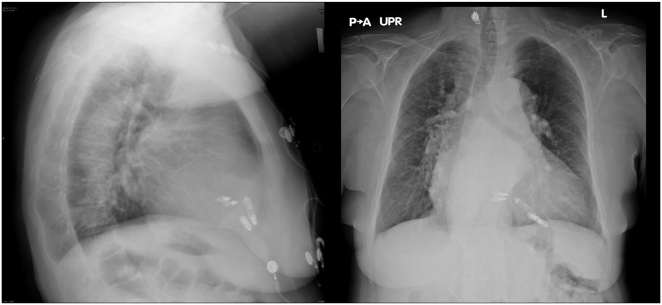
Follow-up X-rays showing parallel placement of both devices.

Follow-up at 1 and 6 months showed normal AVEIR VR parameters: Mode VVI 60 bpm, battery longevity 17.7 years, R-wave 15.3 mV, impedance 690 ohms, capture threshold 0.5 V at 0.4 msec, and >99% ventricular pacing. Holter monitoring (25 hours) confirmed 100% paced rhythm with no oversensing, indicating no device-to-device interaction. Chest X-rays showed parallel device placement (Figure 5).

## Discussion

Two primary strategies exist for managing end-of-life LPMs: implantation of a second device alongside the non-functional one or retrieval followed by new implantation.^6^ Omdahl et al^5^ evaluated RV capacity for multiple Micra devices and concluded that it can accommodate 3 devices without mechanical interaction and with minimal RV volume reduction. Jung et al^7^ reported successful secondary implantation of LPM next to an abandoned one.

Worldwide data on LPM retrieval vary. Afzal et al^8^ reported a 100% retrieval success rate in 29 Micra LPMs implanted for durations ranging from 1 to 95 days, demonstrating that early retrieval is generally feasible with available tools and techniques. Conversely, Lakkireddy et al^9^ reported on retrieval attempts of 73 Nanostim LPMs implanted for durations ranging from 0.2 to 4 years, achieving a slightly lower success rate of 90.4%. This finding suggests that prolonged implant duration may contribute to increased procedural complexity. Additionally, among 115 patients in whom Nanostim devices were abandoned prior to implantation of a new LPM, no adverse device-to-device interactions were observed, thereby reinforcing the safety of leaving non-functional LPMs in situ under selected conditions.^9^ However, the decision to abandon an LPM comes with potential risks, including mechanical interference, device migration, and unpredictable electrical interactions. Frazer et al^10^ highlighted leadless-to-leadless PM interactions, stressing the importance of thorough monitoring in scenarios involving multiple devices.^10^ In this case, multimodal imaging, device interrogation, and Holter monitoring demonstrated no device-to-device interaction, yet ongoing long-term surveillance remains crucial.

The long-term feasibility of retrieving chronically implanted LPMs beyond 5 years remains largely unexamined. One reported case in this context comes from Neužil et al,^11^ who successfully retrieved a helix-fixation LPM at 9 years post-implant in an 84-year-old woman with atrial fibrillation without complications. Retrieval of LPMs is influenced by several factors, including fixation mechanism, implant site, duration, and degree of encapsulation.^12^^,^^13^ Further research is necessary to assess the safety and feasibility of retrieving chronically implanted LPMs beyond 5 years, especially in patients with TVs repaired using TEER, where procedural challenges remain largely unexamined.

In our case, during AVEIR VR manipulation within the RV, the patient experienced a transient 5-second asystole, which resolved spontaneously as Micra VR resumed pacing before the transcutaneous backup was required. Given Micra VR’s stable ventricular pacing at 60 bpm and AVEIR VR’s preset pacing at 40 bpm during implantation, transvenous backup pacing was not initially planned. Introducing a temporary transvenous wire would have increased procedural complexity and posed additional risks, particularly in a patient with a repaired TV and multiple implanted devices. Had the asystole persisted, we were prepared to activate AVEIR VR pacing or, if necessary, implant a transvenous pacing lead.

Micra AV provides atrioventricular (AV) synchrony using an accelerometer-based mechanical atrial sensing system, detecting atrial contractions to optimize ventricular pacing timing. However, this technology depends on effective mechanical transmission of atrial activity, which can be altered by structural modifications to the TV.^14^^,^^15^ In this patient, the presence of TV clips post-TEER and moderate TR raised concerns regarding Micra AV’s ability to reliably track atrial activity. Tricuspid clips may dampen atrial motion, while moderate TR introduces regurgitant flow, further disrupting atrial contraction dynamics and mechanical signal transmission to the leadless device in the RV. Given these limitations, AVEIR VR was selected over Micra AV, providing predictable pacing reliability and longer battery longevity, ensuring consistent device performance despite structural valve modifications.

Future advancements in dual-chamber leadless pacing present a potential upgrade path for this patient. Abbott’s dual-chamber LPM system (AVEIR DR) offers direct atrial-ventricular communication using implant-to-implant (i2i™) technology, bypassing the limitations of mechanical sensing.^16^ With customizable AV delay settings and >95% AV synchrony maintenance across different postures, AVEIR DR represents a future pacing optimization option for our patient with structural heart disease and altered atrial hemodynamics.

## Conclusion

To our knowledge, this is the first reported case of AVEIR VR implantation beside an abandoned Micra VR in a patient with a TV repaired by TEER. This case demonstrates that multiple LPMs can be safely placed in the RV with appropriate imaging guidance, even in the presence of a repaired TV. Future strategies should define the safety and feasibility of retrieving chronically implanted LPMs beyond 5 years through TEER.

## Declaration of generative AI and AI-assisted technologies in the writing process

During the revision of this work, the author(s) used (COPILOT) in order to improve language and readability. After using this tool/service, the author(s) reviewed and edited the content as needed and take(s) full responsibility for the content of the publication.

## Disclosures

The authors have no conflicts of interest to disclose.

## References

[1] Sperzel J., Burri H., Gras D. (2015). State of the art of leadless pacing. Europace.

[2] Adukauskaite A., Hintringer F., Dichtl W., Müller S. (2020). Implantation of leadless pacemaker through neo-orifice after tricuspid valve edge-to-edge repair. Europace.

[3] Jia K., Lampert J., Miller M.A. (2023). PO-04-016 First report of leadless pacemaker implantation in a patient with both a transcatheter tricuspid valve repair (TriClip) and surgical tricuspid valve annuloplasty. Heart Rhythm.

[4] Chen K., Zheng X., Dai Y. (2016). Multiple leadless pacemakers implanted in the right ventricle of swine. Europace.

[5] Omdahl P., Eggen M.D., Bonner M.D., Iaizzo P.A., Wika K. (2016). Right ventricular anatomy can accommodate multiple Micra transcatheter pacemakers. Pacing Clin Electrophysiol.

[6] Beurskens N.E., Tjong F.V., Knops R.E. (2017). End-of-life management of leadless cardiac pacemaker therapy. Arrhythm Electrophysiol Rev.

[7] Jung W., Sadeghzadeh G., Jäckle S., Roggenbuck-Schwilk B., Zvereva V., Kohler J. (2018). Successful implant of a leadless pacemaker with tine-based fixation next to an abandoned battery-depleted screw-in helix fixation leadless device. Europace.

[8] Afzal M.R., Daoud E.G., Cunnane R. (2018). Techniques for successful early retrieval of the Micra transcatheter pacing system: a worldwide experience. Heart Rhythm.

[9] Lakkireddy D., Knops R., Atwater B. (2017). A worldwide experience of the management of battery failures and chronic device retrieval of the Nanostim leadless pacemaker. Heart Rhythm.

[10] Frazer M., Phan F., Dalouk K., Zarraga G.I., Raitt M., Jessel M.P. (2023). A case of leadless-to-leadless pacemaker interaction. HeartRhythm Case Rep.

[11] Neužil P., Petrů J., Chovanec M., Hála P., Šedivá L., Reddy V.Y. (2023). Retrieval and replacement of a helix-fixation leadless pacemaker at 9 years post-implant. HeartRhythm Case Rep.

[12] Li J., Hou W.B., Cao M.K. (2019). Safety and efficacy of leadless pacemaker retrieval. J Cardiovasc Electrophysiol.

[13] González Villegas E., Al Razzo O., Silvestre García J., Mesa García J. (2018). Leadless pacemaker extraction from a single-center perspective. Pacing Clin Electrophysiol.

[14] Garweg C., Willems R. (2024). Advancements in leadless pacemakers: what the second-generation Micra AV2 brings to cardiac care. Touch Cardiogr.

[15] Garweg C., Breitenstein A., Clémenty N. (2024). Strategies to improve atrioventricular synchrony in patients with a Micra AV leadless pacemaker. Europace.

[16] Unveiling the i2i™ communication technology in the AVEIR™ DR leadless pacemaker system. Abbott Cardiac Rhythm Management. https://www.cardiovascular.abbott/us/en/hcp/products/cardiac-rhythm-management/blog/unveiling-the-i2i-communication-technology-in-aveir-dr.html.

