# Supplementary material for: Implantation of leadless pacemaker beside abandoned end-of-life leadless pacemaker in a patient with tricuspid transcatheter edge-to-edge repair
Source: HeartRhythm Case Rep. 2025 Aug 6;11(10):1102–5. doi: 10.1016/j.hrcr.2025.07.028 (PMC12666963; doi:10.1016/j.hrcr.2025.07.028)
Supplement: Supplementary Video Legends [file mmc3.docx]

Video legends

Video 1: Fluoroscopic contrast image displaying the positioning of the AVIER VR leadless pacemaker at the left anterior oblique (LAO) projection prior to deployment.

Video 2: Fluoroscopic contrast image displaying the positioning of the AVIER VR leadless pacemaker at the right anterior oblique (RAO) projection prior to deployment.
